# Deep learning identifies robust gender differences in functional brain organization and their dissociable links to clinical symptoms in autism

**DOI:** 10.1192/bjp.2022.13

**Published:** 2022-02-15

**Authors:** Kaustubh Supekar, Carlo de los Angeles, Srikanth Ryali, Kaidi Cao, Tengyu Ma, Vinod Menon

**Affiliations:** Department of Psychiatry & Behavioral Sciences, Stanford University, USA; Department of Psychiatry & Behavioral Sciences, Stanford University, USA; Department of Psychiatry & Behavioral Sciences, Stanford University, USA; Department of Computer Science, Stanford University, USA; Department of Computer Science, Stanford University, USA; Department of Psychiatry & Behavioral Sciences, Stanford University, USA; Department of Neurology & Neurological Sciences, Stanford University, USA and Wu Tsai Neurosciences Institute, Stanford University, USA

**Keywords:** Autism, explainable artificial intelligence, motor network, reproducible science, clinical heterogeneity

## Abstract

**Background:**

Autism spectrum disorder (ASD) is a highly heterogeneous disorder that affects nearly 1 in 189 females and 1 in 42 males. However, the neurobiological basis of gender differences in ASD is poorly understood, as most studies have neglected females and used methods ill-suited to capture such differences.

**Aims:**

To identify robust functional brain organisation markers that distinguish between females and males with ASD and predict symptom severity.

**Method:**

We leveraged multiple neuroimaging cohorts (ASD *n* = 773) and developed a novel spatiotemporal deep neural network (stDNN), which uses spatiotemporal convolution on functional magnetic resonance imaging data to distinguish between groups.

**Results:**

stDNN achieved consistently high classification accuracy in distinguishing between females and males with ASD. Notably, stDNN trained to distinguish between females and males with ASD could not distinguish between neurotypical females and males, suggesting that there are gender differences in the functional brain organisation in ASD that differ from normative gender differences. Brain features associated with motor, language and visuospatial attentional systems reliably distinguished between females and males with ASD. Crucially, these results were observed in a large multisite cohort and replicated in a fully independent cohort. Furthermore, brain features associated with the motor network’s primary motor cortex node predicted the severity of restricted/repetitive behaviours in females but not in males with ASD.

**Conclusions:**

Our replicable findings reveal that the brains of females and males with ASD are functionally organised differently, contributing to their clinical symptoms in distinct ways. They inform the development of gender-specific diagnoses and treatment strategies for ASD, and ultimately advance precision psychiatry

## Gender differences in autism

Autism spectrum disorder (ASD) is a pervasive and highly heterogeneous neurodevelopmental condition. It is becoming increasingly apparent that systematic disentangling of this high heterogeneity is crucial for developing more precise diagnosis and targeted treatment strategies for ASD. Gender is a key source of heterogeneity in ASD. Indeed, one of the most consistent findings of epidemiological research is that ASD is diagnosed less frequently in females than in males, with a ratio of 1 to 4.^1^ Additionally, converging evidence from behavioural studies suggests that females are less severely affected, especially in the restricted and repetitive behaviours (RRB) symptom domain, than males.^2^ Yet, there is limited research examining the neurobiological differences between females and males with ASD.^3,4^ Additionally, how gender differences in neurobiology relate to gender differences in the clinical symptomatology of the disorder is also not known.^3,4^ Such knowledge is critical both for understanding the aetiology of this heterogeneous disorder and for determining neuroprotective mechanisms in females.^5^ These knowledge gaps are partly because extant brain imaging studies have primarily focused on males and neglected females or used mixed samples involving a small number of females,^3,4^ making it difficult to assess gender-related effects with adequate statistical power. Furthermore, extant studies have relied on conventional univariate approaches that are ill-equipped to capture robust neurobiological gender differences, necessitating the development of new computational approaches.^3^

## Study aims

The first aim of our study was to determine whether neurobiology, in particular functional brain organisation, differed between females and males with ASD. The number of studies examining gender differences in ASD at the brain level is minimal,^4^ and the findings from these studies have been largely inconsistent: some studies have reported gender differences in functional brain organisation in ASD, whereas others have not found any.^3,4^ Critically, the findings from these studies remain poorly replicated, likely because of the small numbers of participants, especially female participants.^3,4^

To address this, we examine, to the best of our knowledge, one of the largest functional brain imaging data-sets to date of females and males with ASD obtained from multiple sites across the world—Autism Brain Imaging Data Exchange (ABIDE)^6^—along with the data we have collected,^7^ as well as an independent Child Mind Institute-Health Brain Network (CMI-HBN) cohort,^8^ using a novel explainable artificial intelligence^9^ (XAI)-based framework.

**Fig. 1:**
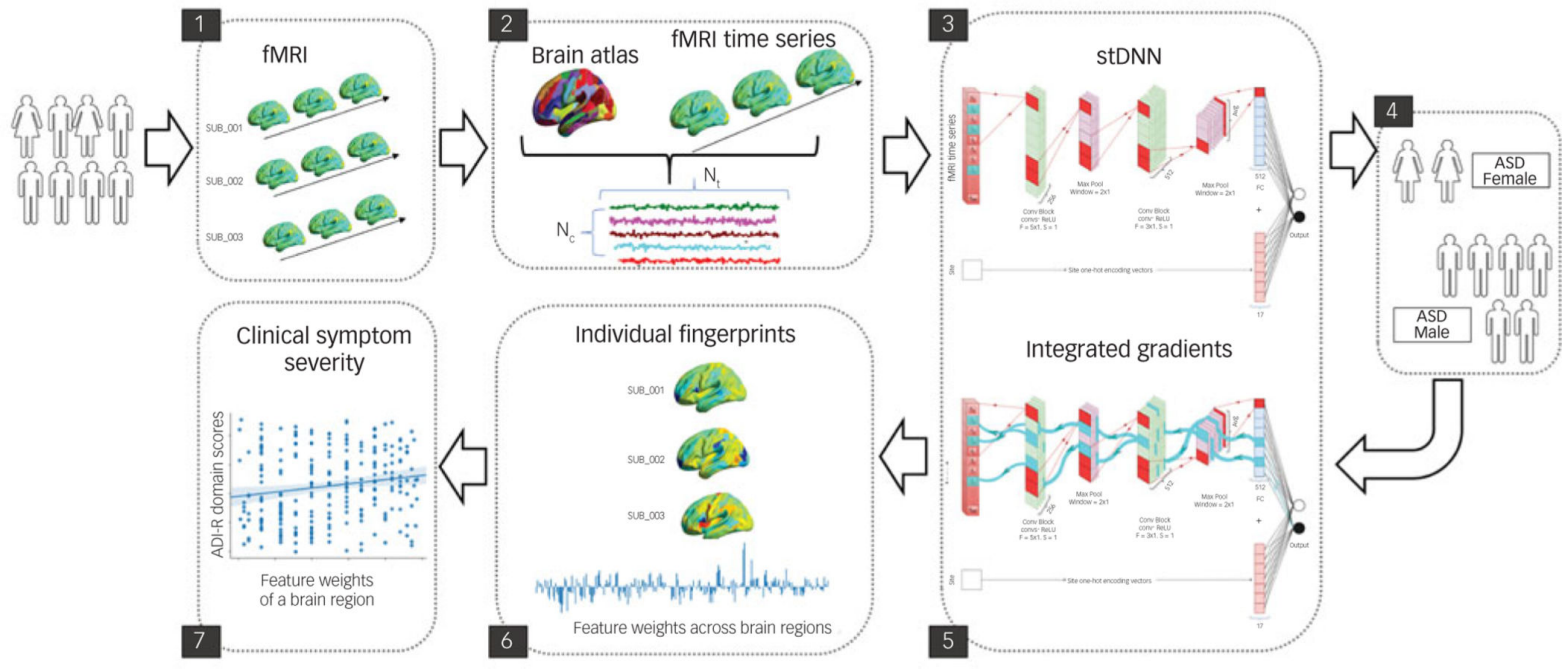
Schematic overview of multicomponent explainable artificial intelligence (XAI) framework for discovering neurobiological patterns/ fingerprints that distinguish between females and males with autism spectrum disorder (ASD) and predict the severity of clinical symptoms. Key steps include: Steps 1, 2: data extraction; Step 3, 4: classification; Steps 5, 6: feature identification. i.e. predictive feature weights (‘fingerprints’) across brain regions; and Step 7: prediction of clinical symptom severity. ADI-R, autism diagnostic interview-revised; Avg, average; F, filter; fMRI, functional magnetic resonance imaging; Nc, number of brain regions; Nt, number of time points; ReLU, rectified linear unit; S, stride; stDNN, spatiotemporal deep neural network.

The field of XAI in recent years has been revolutionised by deep neural networks (DNNs).^10^ DNNs, however, have been far less successful in classification/differentiation of groups using functional brain imaging data.^11^ In fact, no study has employed DNNs to differentiate between females and males with ASD using functional brain imaging data.^3,4^ This is because of the many challenges associated with applying DNNs to brain imaging data (see Supplementary Introduction available at https://doi.org/10.1192/bjp.2022.13 for details). To address these challenges, we developed a novel spatiotemporal DNN (stDNN) model, which takes as its input functional magnetic resonance imaging (fMRI) time series data from brain regions of interest and models the underlying dynamic spatiotemporal characteristics of brain activity to differentiate between females with ASD and males with ASD (Fig. 1 and Supplementary Fig. 1).

A key idea of our approach is to discover latent spatiotemporal dynamics for classification from brain data without the need for explicit feature engineering. Another novel feature of our stDNN model is the use of a one-hot encoding scheme that addresses multisite heterogeneity in fMRI data that is common to data-sharing consortia such as ABIDE. The one-hot encoding scheme enabled the creation of a single stDNN model that handles heterogeneous data while learning robust representations for simultaneously classifying and identifying robust neurobiologically meaningful features that distinguish females and males with ASD. Yet another novel aspect of our stDNN is its use of a label-distribution-aware margin (LDAM) loss^12^ during model training. DNNs fare poorly when the data-set suffers from heavy class imbalance, such as in the present case owing to well-known male bias of ASD.^1^ To improve DNN performance in such scenarios, we designed a theoretically principled LDAM loss motivated by minimising a marginbased generalisation bound, which we have previously shown to outperform the conventional cross-entropy loss under class-imbalance conditions.^12^

We applied our stDNN model with LDAM loss function to the multisite ABIDE^6^ combined with Stanford^7^ task-free fMRI data. We hypothesised that stDNN would be able to accurately distinguish between females and males with ASD in the ABIDE/Stanford cohort.

To address growing concerns about reproducibility in neuroscience, we next applied our stDNN model with LDAM loss to CMI-HBN cohort task-free fMRI data.^8^ It should be noted that the CMI-HBN cohort data was not used for training the stDNN model, and therefore it is a fully independent data-set for demonstrating the generalisability of the stDNN ASD gender classification model. This is a crucial step in which most approaches are widely known to fail.^11^

We hypothesised that stDNN trained on ABIDE/Stanford cohort would be able to accurately distinguish between females and males with ASD in the fully independent CMI-HBN cohort. To determine the specificity of gender differences in functional brain organisation in ASD, we assessed whether the stDNN model trained to distinguish between females and males with ASD could also distinguish between neurotypical females and neurotypical males. We further hypothesised that the stDNN ASD gender classification model would not be able to accurately distinguish between neurotypical females and neurotypical males, reflecting unique patterns of gender differences in ASD.^2^

The second aim of our study was to determine which aspects/ features of functional brain organisation differed between females and males with ASD. Conventional DNN approaches are black box models, which provide no insight into which brain features are important for classification, nor whether the features are neurobiologically interpretable in the context of previous research of gender differences in ASD.^11^ We address this gap by using an integrated gradients method^13^ for identifying neurobiologically meaningful features that distinguish between females and males with ASD. This method ranks brain features that distinguish between females and males with ASD. We hypothesised that integrated gradients would reveal functional organisation patterns that are different between females and males with ASD in multiple brain areas, particularly those belonging to the motor and language networks.^2^ We further predicted that the patterns of ASD gender differences in functional brain organisation would differ from the normative/ typical gender difference patterns.^2^

The third aim of our study was to examine the relationship between the functional brain organisation patterns that differ in females and males with ASD and their symptom severity. To our knowledge, no previous study has examined functional brain features that robustly predict clinical symptoms in females and males with ASD separately without using feature engineering. We hypothesised that the brains of females and males with ASD would be functionally organised in ways that contribute differently to their clinical symptoms.^2^

## Method

### Participants

#### ABIDE

We leveraged neuroimaging and phenotypic data from ABIDE^6^ (Supplementary Table 1, Supplementary Fig. 2, see Supplementary Methods for details).

#### Stanford

An independent cohort of participants recruited and scanned at Stanford University^7^ was used to further increase the number of females with ASD in our sample (Supplementary Table 1, Supplementary Fig. 3, see Supplementary Methods for details).

Written informed consent was obtained from the participant’s legal guardian. The study protocol was approved by the Stanford University Institutional Review Board. Specifically, the ABIDE data was combined with the Stanford data to form the ABIDE/ Stanford cohort, which served as the primary cohort.

#### CMI-HBN

An independent cohort of participants from CMI-HBN^8^ was used to demonstrate the robustness of our findings. (Supplementary Table 2, Supplementary Fig. 4, see Supplementary Methods for details).

### stDNN model

We developed an innovative stDNN model to extract informative functional brain dynamics features that accurately distinguish between females and males with ASD (see Supplementary Methods for details). Briefly, our stDNN model consists of two 1D-convolutional block layers, a ‘temporal averaging’ operation, and then a sigmoid output layer (Supplementary Fig. 1). Pre-processed regional fMRI time series from 246 brain regions defined in the Brainnetome atlas were given as input to the first 1D-convolutional block layer. To account for site-related heterogeneity, site information encoded with a one-hot encoding scheme was given as an input to the final layer. stDNN classified participants in the two groups by minimising a LDAM loss function.

### Fivefold cross-validation ASD gender classification analysis of ABIDE/Stanford cohort data

**Fig. 2:**
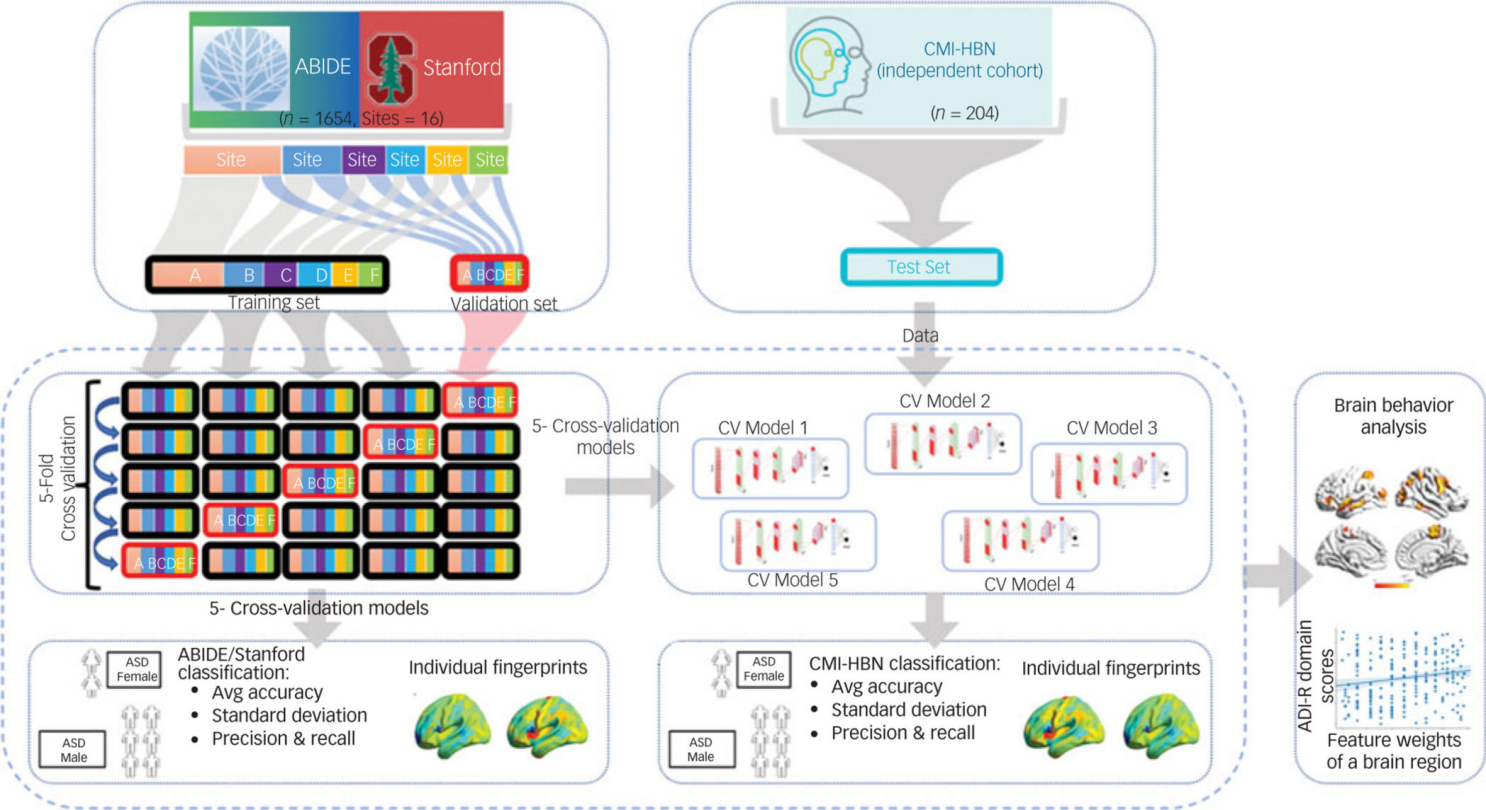
Fivefold cross-validation procedure for testing and validation of females with autism spectrum disorder (ASD) versus males with ASD classification using the ABIDE/Stanford cohort. The five models are then used for independently testing females with ASD versus males with ASD classification in the Child Mind Institute-Health Brain Network (CMI-HBN) cohort. Note that these models are not trained on the CMI-HBN cohort. ADI-R, autism diagnostic interview-revised; Avg, average; CV, cross-validation.

To prevent bias and account for low variance, we conducted a fivefold cross-validation to evaluate the performance (accuracy, precision, recall, and the harmonic mean of precision and recall (F1)) of our stDNN model in classifying ASD females versus ASD males (Fig. 2; see Supplementary Methods for details).

### ASD gender classification analysis of CMI-HBN cohort data using fivefold ABIDE/Stanford cohort ASD gender classification models

For reporting the performance of our stDNN for the CMI-HBN cohort, we used each of the five stDNN models trained on different subsets of the ABIDE/Stanford cohort (Fig. 2; see Supplementary Methods for details).

### Neurotypical gender classification analysis of ABIDE/ Stanford cohort data using fivefold ABIDE/Stanford cohort ASD gender classification models

To examine the specificity of our stDNN ASD gender classification model, we investigated whether the stDNN model trained to distinguish between females with ASD and males with ASD can distinguish between neurotypical females and neurotypical males in the ABIDE/Stanford cohort (see Supplementary Methods for details).

### Fivefold cross-validation neurotypical gender classification analysis of ABIDE/Stanford cohort data

We conducted a fivefold cross-validation to evaluate the performance (accuracy, precision, recall, F1) of our de novo trained stDNN model in classifying neurotypical females versus neurotypical males (see Supplementary Methods for details).

### Identifying brain features underlying ASD gender classification

We used an integrated gradients-based feature attribution approach^13^ (see Supplementary Methods for details) to identify brain features that discriminated between females with ASD and males with ASD.

### Clinical symptom prediction in females and males with ASD

We investigated the relationship between stDNN-identified neurobiological features (that distinguished between females and males with ASD) with the severity of clinical symptoms in females with ASD and males with ASD separately. Spearman correlations between the autism diagnostic interview-revised (ADI-R)^14^ domain scores and the brain features derived from each of the five stDNN models were computed (see Supplementary Methods for details).

### Control analyses

We performed several control analyses to demonstrate that our findings are robust to head motion-related confounds (see Supplementary Methods for details).

## Results

### Classification of ASD females versus ASD males in the ABIDE/Stanford cohort

We first trained our stDNN on the multisite ABIDE cohort data combined with data we acquired at Stanford (ASD cohort *n* = 678) (Supplementary Table 1). To assess the performance of our stDNN model, we used a fivefold cross-validation procedure in which 80% of the sample was used for training and the other 20% of the sample was used for validation (Fig. 2). stDNN achieved an average accuracy of 86.0% (s.d. = 1.65%) across the five folds, and an average precision of 0.86 (s.d. = 0.02), recall of 0.86 (s.d. = 0.02) and F1 score of 0.83 (s.d. = 0.02) (Supplementary Table 3). Additional analyses confirmed that the observed results were robust to potential confounds such as head motion (see Supplementary Results for details). These results demonstrate that stDNN can accurately distinguish females with ASD from males with ASD in a multisite cohort, and, furthermore, does so in a robust and consistent manner across cross-validation folds.

### Classification of ASD females versus ASD males in an independent CMI-HBN cohort

We then evaluated the performance of our stDNN model on an independent cohort of females with ASD and males with ASD obtained from CMI-HBN (ASD cohort *n* = 95) (Supplementary Table 2). Importantly, the stDNN was not trained on the CMI-HBN data. We evaluated five models corresponding to each of the folds in the cross-validation as described above (Fig. 2). stDNN achieved an average accuracy of 83.4% (s.d. = 3.67%) across the five folds, and an average precision of 0.85 (s.d. = 0.01), recall of 0.83 (s.d. = 0.04) and F1 score of 0.84 (s.d. = 0.02) (Supplementary Table 4). These results demonstrate that stDNN can accurately distinguish females with ASD from males with ASD in an independent cohort without additional training.

### Classification of neurotypical females versus neurotypical males

To examine the specificity of our stDNN ASD gender classification model, we investigated whether the stDNN model trained to distinguish between females with ASD and males with ASD can distinguish between neurotypical females and neurotypical males in the ABIDE/ Stanford cohort (neurotypical cohort *n* = 976) (Supplementary Table 1). For the neurotypical females versus neurotypical males classification, the stDNN model trained on the ABIDE/Stanford ASD data achieved an accuracy of 66.7% (s.d. = 0.75%) across the five folds, and an average precision of 0.64 (s.d. = 0.01), recall of 0.67 (s.d. = 0.01) and F1 score of 0.60 (s.d. = 0.02) (Supplementary Table 5).

These classification metric values were much lower than those obtained by a *de novo* trained stDNN model on ABIDE/Stanford neurotypical data, which achieved an accuracy of 77.8% (s.d. = 0.38%), across the five folds, and an average precision of 0.78 (s.d. = 0.01), recall of 0.77 (s.d. = 0.01) and F1 score of 0.78 (s.d. = 0.02) (Supplementary Table 6).

These results point to a unique pattern of gender differences in ASD.

### Identification of brain features underlying ASD gender classification in the ABIDE/Stanford cohort

**Fig. 3:**
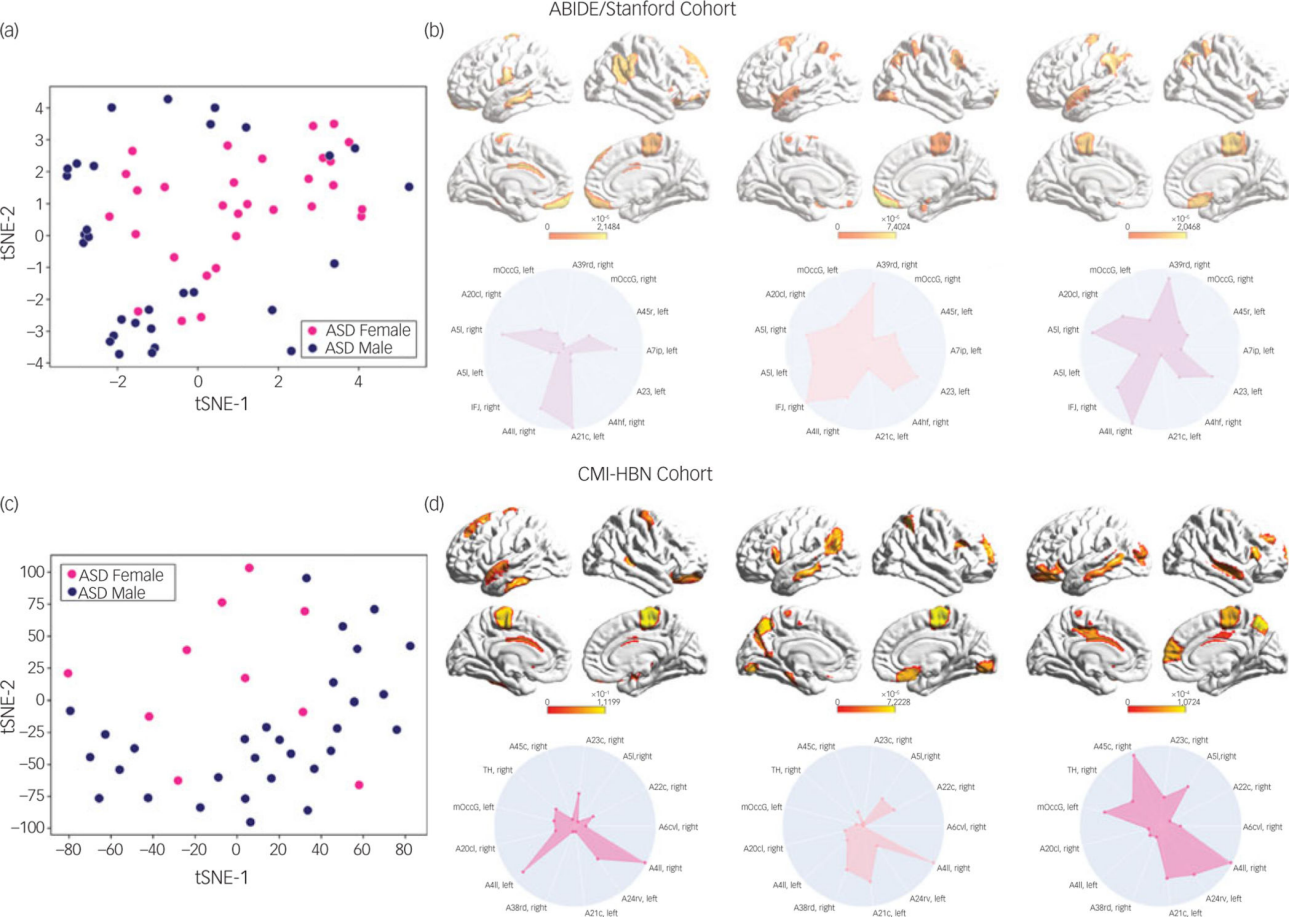
(a) t-distributed stochastic neighbour embedding (tSNE) plot of spatiotemporal deep neural network (stDNN)-derived individual feature attribution maps/fingerprints of 30 representative females with ASD and 30 representative males with ASD from the ABIDE/Stanford cohort, demonstrating the clustering of females with ASD and males with ASD. (b) stDNN-derived individual feature attribution maps/fingerprints in three females with ASD from the ABIDE/Stanford cohort. (c) tSNE plot of stDNN-derived individual feature attribution maps/fingerprints of 10 representative females with ASD and 30 males with ASD from the Child Mind Institute-Health Brain Network (CMI-HBN) cohort, demonstrating the clustering of females with ASD and males with ASD. (d) stDNN-derived individual feature attribution maps/fingerprints in three females with ASD from the CMI-HBN cohort.

We then used an integrated gradients procedure^13^ to compute the feature attributes underlying the ASD female class label in the ABIDE/Stanford cohort. This analysis yields a measure of feature strength associated with females with ASD versus males with ASD classification in each brain region and at each time point. The integrated gradients procedure was applied to the stDNN model that is trained to distinguish between females with ASD and males with ASD using ASD data from the ABIDE/Stanford cohort. This procedure also identifies an individual fingerprint of predictive features in each participant (Fig. 3, see Supplementary Results for details).

**Fig. 4:**
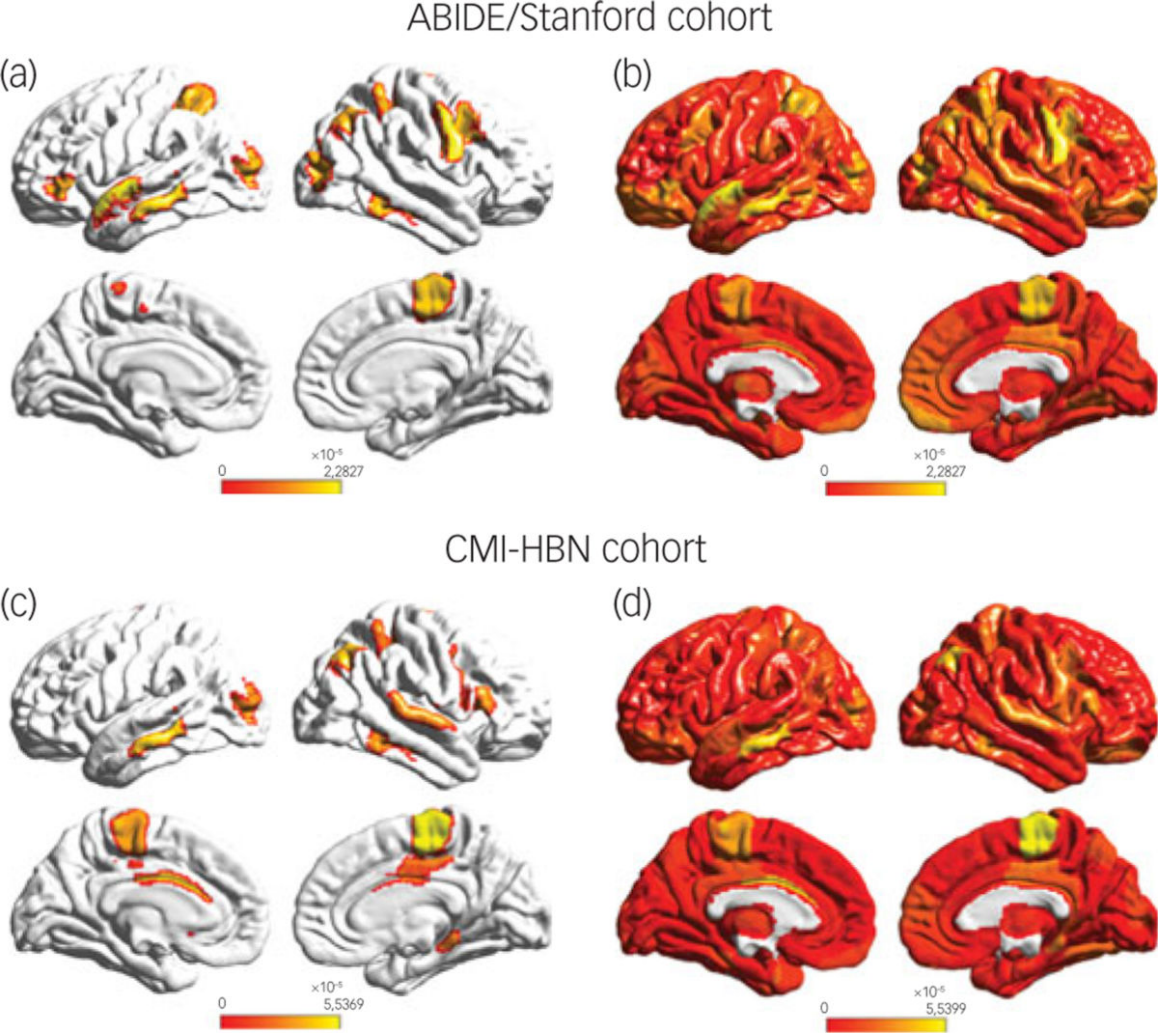
(a) Feature attribution map showing the top 5% features that underlie females with ASD versus males with autism spectrum disorder (ASD) classification in the ABIDE/Stanford cohort. Spatiotemporal deep neural network (stDNN) with integrated gradients identified brain features that distinguish females with ASD from males with ASD. The algorithm automatically identified distinguishing features in the primary motor cortex and the supplementary motor area, which anchor the motor network, middle and superior temporal gyri, which anchor the language network, as well as the visuospatial attentional system (see Supplementary Table 7 for a detailed listing of brain areas and predictive feature weights). (b) Visualisation of (unthresholded) feature weights across the whole brain in the ABIDE/Stanford cohort. (c) Feature attribution map showing the top 5% features showing replication of predictive motor network, language network, and visuospatial attention features in the Child Mind Institute-Health Brain Network (CMI-HBN) cohort (see Supplementary Table 8 for a detailed listing of brain areas and predictive feature weights). (d) Visualisation of (unthresholded) feature weights across the whole brain in the CMI-HBN cohort.

To identify brain areas that contributed the most to classification, we computed the median of feature scores across the five folds and thresholded them – top 5% of features – based on the distribution of feature scores across all time points and regions. This resulted in the identification of a distributed set of brain areas including the primary motor cortex, supplementary motor area, parietal and lateral occipital cortex, and middle and superior temporal gyri as brain areas that contribute most significantly to predicting the ASD female class label (Fig. 4 and Supplementary Table 7). These brain areas were distinct from those that contributed most significantly to predicting the neurotypical female class label (see Supplementary Results for details).

Additional analyses confirmed that the observed results were robust to potential confounds such as head motion (see Supplementary Results for details). These results demonstrate that stDNN together with integrated gradients procedures automatically identifies discriminating features without the need for *ad hoc* feature engineering procedures.

### Identification of brain features underlying ASD gender classification in the CMI-HBN cohort

We then used the same procedures as described in the previous section to determine predictive feature attributes in each female participant with ASD in the CMI-HBN cohort. This analysis revealed individualised brain ‘fingerprints’ (Fig. 3, see Supplementary Results for details), and identified the primary motor cortex, supplementary motor area, parietal and lateral occipital cortex, and middle and superior temporal gyri as the brain areas that contributed most significantly to predicting the ASD female class label (Fig. 4 and Supplementary Table 8).

These results demonstrate that stDNN together with integrated gradients procedures automatically identifies similar discriminating features as in the ABIDE/Stanford cohort, again without the need for *ad hoc* feature engineering procedures.

### Predicting clinical symptoms using brain features

We investigated whether stDNN-identified brain features could predict the severity of clinical symptoms in females and males with ASD. The primary motor cortex was the only brain region whose features predicted ADI-R RRB scores (*P* < 0.01; false discovery rate-corrected) in females with ASD. No such relationship was observed in males with ASD or for the social and communication domains in either females or males with ASD, demonstrating the specificity of findings related to RRB phenotypic features in females with ASD.

## Discussion

### Main findings

By using one of the largest functional brain imaging cohorts of females and males with ASD and leveraging exciting recent advances in XAI, we examined neurobiological gender differences in ASD. To our knowledge, this is the first use of a XAI-based approach for uncovering robust neurobiological gender differences in ASD. Our XAI-based approach is also a significant advance over previous approaches to find gender differences in ASD using functional brain imaging data (see Supplementary Discussion for details). Our novel XAI-based stDNN model, which uses spatiotemporal convolution on fMRI data to distinguish between groups, achieved consistently high classification accuracy in distinguishing between females and males with ASD. Notably, the stDNN model trained to distinguish between females and males with ASD could not distinguish between neurotypical females and males, suggesting that there are gender differences in the functional brain organisation in ASD and these gender differences are different from normative gender differences.

Brain features associated with motor, language and visuospatial attentional systems reliably distinguished females with ASD from males with ASD. Crucially, these results were observed in the large multisite ABIDE/Stanford cohort and replicated in a fully independent CMI-HBN cohort. Furthermore, brain features associated with the primary motor cortex node of the motor network emerged as a robust predictor of the severity of RRBs in females with ASD but not in males with ASD. Taken together, these results, elaborated below, provide novel and robust insights into the neurobiology of gender differences in ASD and their relation to the core clinical symptoms of the disorder.

### Interpretation of our findings

The first key finding of our study is that females with ASD have a functional brain organisation that differs significantly from males with ASD. Gender differences in functional brain organisation in ASD reported to date have been mixed and, to the best of our knowledge, no studies have attempted model-based validation and replication of findings. stDNN allowed us to perform cross-validation analysis, a powerful approach for validating research findings, and its use for demonstrating generalisation and reproducibility is now increasingly advocated in psychiatry.^15^ Notably, stDNN also allowed us to develop a model based on one data-set (ABIDE/Stanford) and test it on another ‘independent’ data-set (CMI-HBN) without further training. This is a crucial step in which most approaches in other domains are widely known to fail.^15^

Our stDNN model learned functional brain organisation patterns that could distinguish between females and males with ASD with a high cross-validation classification accuracy in the multisite ABIDE/Stanford cohort, despite the considerable heterogeneity in data acquisition protocols and a wide range in age (mean age = 13.2 years, s.d. = 5.9) and symptom profiles. Notably, stDNN achieved a high classification accuracy in an independent CMI-HBN cohort (mean age = 11.8 years, s.d. = 3.9) that was not used in the training of stDNN. These results further demonstrate robust gender differences in functional brain organisation in ASD.

stDNN also enabled us to seamlessly determine whether functional brain patterns that distinguished between females and males with ASD could also classify by gender in data from neurotypical peers. We found that the stDNN model trained to distinguish between females and males with ASD could not distinguish between neurotypical females and males. These results point to unique neurofunctional patterns of gender differences in ASD.

The second main finding of our study is the identification of functional brain features that distinguish between females and males with ASD. Our stDNN-based integrated gradients analysis identified the primary motor cortex and supplementary motor area, which anchor the motor network, as brain areas whose dynamical functional properties most clearly distinguished between females and males with ASD in both cohorts. Aberrancies in the extended motor network have been consistently reported in ASD, when compared with neurotypical individuals.^16^ An open unaddressed question is whether these same brain areas also show gender-specific differentiation, which our findings help resolve in a robust manner across multiple cohorts. This question is important because aberrancies in key nodes of the motor network and their dynamic functional interactions have been linked to fine and gross motor deficits in ASD,^16^ and there is increasing evidence for gender differences in motor deficits in ASD.^17^

Previous studies have reported ASD-related gender differences in grey matter morphometry of the motor network, albeit using small sample sizes.^2^ Our findings, using a large sample of females and males with ASD, extend these results by providing novel and robust evidence that the functional brain organisation of the motor system, which has been consistently shown to differ between ASD and their neurotypical peers, is also different between females and males with ASD.

In both cohorts, our analysis also identified bilateral middle and superior temporal gyri, which anchor the language system, as brain areas whose features clearly distinguished between females and males with ASD. Impairments in temporal cortex areas associated with language processing are a prominent feature of ASD.^18,19^ Language systems anchored in the middle and superior temporal gyri facilitate speech processing and semantic comprehension, processes known to be impaired in ASD.^20^

Surprisingly, our analysis also identified the dorsal parietal cortex as a brain area whose features distinguished between females and males with ASD in both cohorts. Specific loci included the superior parietal lobule and the adjoining posterior human intraparietal area 3 (hIP3) subdivision of the intraparietal sulcus, which play a crucial role in visuospatial attention.^21,22^ Moreover, these parietal areas, encompassing the dorsal visual stream pathway, have strong connections with the lateral occipital gyrus^21^ whose features also distinguished between females and males with ASD. Together, these regions constitute key elements of the dorsal and ventral visual pathways involved in attending to the location of objects in space.^23^ This finding of gender-related differences is noteworthy because of conflicting evidence in the extant literature about co-occurring parietal and lateral occipital cortex impairments in ASD.^18,19^

Taken together, these results identify gender differences in motor and language systems that are known to be impaired in the disorder as well as dorsal parietal and lateral occipital cortex regions that have not been reported previously in the literature as being affected in ASD. Interestingly, although default mode network and salience network aberrancies have been consistently reported in ASD,^24,25^ gender differences were not reliably observed in these regions, suggesting that aberrancies in the default mode network and salience network may be common to females and males with ASD. More broadly, our findings suggest that brain characteristics of gender differences in ASD overlap only partly with overall brain aberrancies in ASD, emphasising the convergent and distinct underlying mechanisms of gender differences in the disorder.

These observations parallel findings from neuroanatomical studies that suggest that the overall ASD neuroanatomy in females and males does not simply differ quantitatively in the same brain regions/circuitries but also differs qualitatively.^26^ The extent to which abnormalities in the brain systems identified in the present study differentially have an impact on the acquisition of motor, visuospatial and social communication skills in females and males with ASD remains to be investigated.

A third important finding of our study is that we found that the primary motor cortex node of the motor network was the only brain area whose dynamic functional circuitry predicted scores on the RRB domain of the ADI-R. They were not related to the scores on the social and communication scores of the ADI-R, indicating domain-specific effects associated with RRBs – a core clinical phenotype of ASD that has been most consistently reported to differ between genders.^2^ Surprisingly, these relationships were observed in females, but not in males, with ASD. The neurofunctional mechanisms underlying the heterogeneity of RRB symptoms in males with ASD remain to be investigated. A potential avenue for investigation might be to examine the relationship between RRBs in males with ASD and the functional organisation of other components of the motor network, including the basal ganglia and cerebellum, which we recently found to be predictive of repetitive motor behaviours in a predominantly male ASD sample.^7^

### Implications

Our findings reveal that the brains of females and males with ASD are functionally organised differently, contributing to their clinical symptoms in distinct ways. Our discovery of robust neurobiological gender differences in ASD psychopathology has the potential to transform our understanding of the diverse aetiologies of the disorder, as well as inform the development of gender-specific diagnosis and treatment strategies. Our approach, in general, provides new XAI-based tools for investigating the robust neurobiological bases of psychiatric disorders and the accompanying clinical symptoms, with the potential to inform precision psychiatry.

## Supplementary Material

1

## Data Availability

Data that support the findings of this study are openly available in ABIDE and CMI-HBN. The Stanford cohort data examined in the study are available from the corresponding author, K.S., upon reasonable request.
