# Supplementary material for: Deep learning identifies robust gender differences in functional brain organization and their dissociable links to clinical symptoms in autism
Source: Br J Psychiatry. Author manuscript; Available in PMC 2023 Aug 15. (PMC9376194; doi:10.1192/bjp.2022.13)
Supplement: 1 [file NIHMS1771764-supplement-1.docx]

**Deep learning identifies robust sex differences in functional brain organization and their dissociable links to clinical symptoms in autism**

***Supplemental Information***

Contents

Supplemental Introduction pg. 2

Supplemental Methods pg. 4

Supplemental Results pg. 13

Supplemental Discussion pg. 18

Supplemental Tables S1-S8 pg. 21

Supplemental Figures S1-S4 pg. 29

Supplemental References 1-31 pg. 33

Supplementary Introduction

**Challenges associated with applying deep neural networks (DNNs) to brain imaging data**

DNNs have been far less successful in classification/differentiation of groups using functional brain imaging data (1). In fact, no study has employed DNNs to differentiate between females and males with ASD using functional brain imaging data (2, 3). This is due to the many challenges associated with applying DNNs to brain imaging data, most notably dealing with the high dimensionality of the whole brain data as well as noisy measurements with a large degree of individual variability (1).

A few recent studies have used DNNs with explicitly engineered features to reduce the dimensionality of brain data, albeit none of these studies have examined neurobiological sex differences in ASD (1, 4). These studies typically involve first selecting individual brain regions using functional parcellation, from which fMRI time series are extracted to precompute interregional static functional connectivity. Generally, DNN models that use these engineered features consist of several fully connected networks followed by a sigmoid layer for classification. There are two problems with this approach. First, the correlation coefficients, which are used as static functional connectivity features, assume that the fMRI time series reflect a stationary process. Recent studies have revealed, however, that fMRI time series are highly nonstationary (5-7), and linear measures such as correlations can be inadequate to account for them. Second, DNN models with fully connected layers are difficult to train, especially in neuroimaging applications, since there are a large number of free parameters and limited labelled training data. As a result, these architectures tend to overfit the data and do not allow for building deep networks, which are essential for robust performance and out-of-sample prediction (8). Critically, extant approaches fail to exploit the dynamic spatiotemporal characteristics of brain activity that are believed to contain more robust features of psychiatric disorders (9-11).

Supplementary Methods

**Study cohorts**

**ABIDE** We leveraged neuroimaging and phenotypic data from the Autism Brain Imaging Data Exchange (ABIDE; http://fcon_1000.projects.nitrc.org/indi/abide/) (12, 13). The subject selection procedure is illustrated in **Supplementary Figure S2**. **Supplementary Table S1** shows demographic information.

**Stanford** An independent cohort of participants recruited and scanned at Stanford University (14-16) was used to further increase the number of females with ASD in our sample. Written informed consent was obtained from the participant’s legal guardian. The study protocol was approved by the Stanford University Institutional Review Board. Specifically, the ABIDE data was combined with the Stanford data to form the ABIDE/Stanford cohort, which served as the primary cohort. ASD diagnosis was assessed using Autism Diagnostic Observation Schedule (ADOS) and ADI-R as described in our published studies (16-18). The subject selection procedure is illustrated in **Supplementary Figure S3**. **Supplementary Table S1** shows demographic information.

**CMI-HBN.** An independent cohort of participants made available through the CMI-HBN (19) consortium (http://fcon_1000.projects.nitrc.org/indi/cmi_healthy_brain_network/) was used to demonstrate the robustness of our findings. The subject selection procedure is illustrated in **Supplementary Figure S4**. **Supplementary Table S2** shows demographic information.

**fMRI preprocessing**

All functional MRI data were prepared by using SPM12 software package, as well as in-house MATLAB scripts. Structural MRI images were segmented into grey matter, white matter (WM), and cerebrospinal fluid (CSF). Resting-state functional MRI (fMRI) data were realigned to the averaged time frame to correct for head motion, slice-time corrected to the first slice, and co-registered with each participant’s T1-weighted images. The functional images were then normalized to the standard Montreal Neurological Institute (MNI152) template in 2mm3. A 6-mm Gaussian kernel was used to spatially smooth the functional images and a band-pass filter ranging from 0.01 to 0.1 Hz was applied. To account for artifacts from motion and nonneural sources, the mean time series from each of the CSF and WM masks as well as 6 motion parameters, obtained by rigid body registration, were regressed out from the fMRI data.

**Data input into the stDNN**

We used the Brainnetome Atlas (246 regions) (20) and computed the average resting-state fMRI timeseries across the voxels in each region of interest (ROI). Each participant’s time series data was represented by a matrix of size $N_{C}\times N_{T}$ where $N_{C}$ is the number of channels or ROIs and $N_{T}$ is the number of time points. We used site information as a covariate.

**stDNN model**

We developed an innovative stDNN model to extract informative brain dynamics features that accurately distinguish between females with ASD and males with ASD. A key advantage of our approach is that it provides a novel technique to capture latent dynamics without the need for explicit feature engineering(21). Our stDNN model consists of two 1D convolutional block layers, a “temporal averaging” operation, and then a sigmoid layer (**Supplementary Figure S1**). Each convolutional block layer consists of a convolutional operation and ReLU activation. We introduce “maxpool” layers after each of the two convolutional block layers. These “maxpool” layers help in (a) reducing the temporal dimension of the data, (b) hierarchical representation of the features, and (c) increasing the receptive field of the filter to capture the long-term correlations in the timeseries. Conventionally, after the last convolutional block, the data is flattened, and a fully connected layer is connected to the output sigmoid layer. The fully connected layers typically have the maximum number of parameters to be trained compared to the convolutional layers. In our model, instead of the normal flattening operation, we use a static “temporal averaging layer” where we average the temporal features for each filter and therefore the number of inputs to the fully connected layer is just the number of output channels of the second convolution block layer. The advantage of the static temporal averaging layer over the flattening layer: the number of parameters reduces from $N_{C2}\times N_{T2}$ to $N_{C2}$ where $N_{C2}$is the number of output channels of the second convolutional block layer and $N_{T2}$ is the temporal dimension of the output of the second “maxpool” layer. The advantages of the static temporal averaging over a trainable temporal averaging layer: with the static temporal averaging layer, we can train and test fMRI timeseries with varying time lengths. Varying time length is common with open-source data where the data is acquired with different data acquisition protocols. We introduce a dropout layer (= 0.5) before the fully connected layer to avoid overfitting during the model training process. To account for site-related heterogeneity, site information encoded with a one-hot encoding scheme was given as an input to the final fully connected layer. In a one-hot encoding scheme, a categorical variable is converted and provided as an input to a DNN to improve prediction. Specifically, in our case, each site value is converted to a new binary variable column and assigned a value of 0 or 1, resulting in a site feature vector of size 1 by *m*, where *m* is the number of sites in the ABIDE/Stanford cohort. The site feature vector associated with a subject from site *i*, for example, will have a 1 in the *i*-th column of the feature vector and zeroes in all other columns.

We train the model for up to 100 epochs with a stopping criterion and a learning rate of 0.00003 with a batch size of 16. An Adam optimizer with a weight decay of 0.0001 was used to estimate the stDNN model parameters (22). To address the heavy class imbalance in our dataset (number of females << number of males), we used (i) a theoretically principle label-distribution-aware-margin (LDAM) loss (23) instead of the conventional cross-entropy loss, and (ii) class re-weighting. After the initial stages of training, the less frequent class (female) samples were given a higher weight, while the more frequent class (male) samples were assigned a lower weight. We applied re-weighting only at later stages of training to allow the model to learn an initial representation of the data without the influence of complexity associated with class re-weighting. The optimal model parameters where derived and confirmed through hyperparameter tuning using Ray Tune on data from the training fold (24).

**Five-fold cross-validation ASD sex classification analysis of ABIDE/Stanford cohort data**

To prevent bias and account for low variance, we conducted a five-fold cross-validation to evaluate the performance of our stDNN model in classifying ASD females vs ASD males. In the five-fold cross-validation approach, we divided the ABIDE/Stanford ASD dataset into five different parts, where we use four parts for training and validation and the fifth part as the test set. We then rotate through the whole dataset five times to select a different section as the test set during each iteration (**Figure 2).** For each of the five subsets, we evaluate the performance of our stDNN model individually and then average over the five subsets to report the mean and standard deviation values of the key performance metrics (accuracy, precision, recall, F1). Using the five-fold cross-validation approach, the performance for every sample from the ABIDE/Stanford data gets accounted, which helps in assessing the effectiveness of the model more robustly instead of just reporting the performance on one-time random split of the data.

**ASD sex classification analysis of CMI-HBN cohort data using five-fold ABIDE/Stanford cohort ASD sex classification models**

Similar to the five-fold cross-validation process used for the ABIDE/Stanford cohort, for reporting the performance of our stDNN for the CMI-HBN cohort, we use each of the five stDNN models trained on different subsets of the ABIDE/Stanford cohort. Using the five different models, we evaluate each model’s performance on the CMI-HBN cohort data independently (**Figure 2**) and report the mean and standard deviation values of the key performance metrics (accuracy, precision, recall, F1).

**Neurotypical sex classification analysis of ABIDE/Stanford cohort data using five-fold ABIDE/Stanford cohort ASD sex classification models**

To examine the specificity of our stDNN ASD sex classification model, we investigated whether the stDNN model trained to distinguish between females with ASD and males with ASD can distinguish between neurotypical females and neurotypical males in the ABIDE/Stanford cohort. Specifically, we used each of the five stDNN models trained to classify ASD Female vs ASD Male on different subsets of the ABIDE/Stanford cohort. Using the five different models, we evaluate each model’s performance on the ABIDE/Stanford neurotypical data and report the mean and standard deviation values of the key performance metrics (accuracy, precision, recall, F1).

**Five-fold cross-validation neurotypical** **sex classification analysis of ABIDE/Stanford cohort data**

We conducted a five-fold cross-validation to evaluate the performance of our stDNN model in classifying neurotypical females vs neurotypical males. In the five-fold cross-validation approach, we divided the ABIDE/Stanford neurotypical dataset into five different parts, where we use four parts for training and validation and the fifth part as the test set. We then rotate through the whole dataset five times to select a different section as the test set during each iteration**.** For each of the five subsets, we evaluate the performance of our stDNN model individually and then average over the five subsets to report the mean and standard deviation values of the key performance metrics (accuracy, precision, recall, F1). Using the five-fold cross-validation approach, the performance for every sample from the ABIDE/Stanford data gets accounted, which helps in assessing the effectiveness of the model more robustly instead of just reporting the performance on one-time random split of the data

**Identifying brain features underlying ASD sex classification**

We used an integrated gradients (IG)-based feature attribution approach (25-29) to identify brain features that distinguished between ASD females and ASD males. A major problem in developing and evaluating feature attribution methods is that it is difficult to distinguish errors from the DNN model and those from feature attribution procedures. IG solves this problem by taking an approach that satisfies two fundamental axioms – sensitivity and implementation invariance (25-29). Another advantage of IG is that the gradients can be computed easily for any given network architecture. IG estimates the integral of gradients with respect to the *i-th* dimension of the input *x* along the straight-line path from a given (or random) baseline to the input as follows:

$${IG}_{i}=(x-x^{'})\int_{0}^{1} {(x}_{i}-{x^{'}}_{i})\frac{\partial F(x^{'}+ \alpha\left( x-x^{'} \right))}{\partial x_{i}}\partial\alpha$$

where, ${IG}_{i}$ is the integrated gradients for the *i-th* component of the input *x* and $x^{'}$ is the baseline input for which the neural network *F* results in a neutral output. IG provides a score of how important each feature contributes to the final prediction. This approach provides insights about important features that predict ASD class label. Conventional gradient-based approaches wrongly assign zero attributions for inputs where the function is flat, even when the output of *F* for such an input is different from the baseline. IG avoids this problem by computing an average gradient along a linear path. Our IG implementation is based on the *“Captum” (https://captum.ai/docs/introduction.html)* module of *Pytorch.*

IG analysis yields a measure of feature strength associated with ASD females vs ASD males classification in each brain region and at each time point. To identify brain areas that contributed the most to classification, we computed the median of feature scores across the five folds and thresholded them - top 5% of features - based on the distribution of feature scores across all time points and regions. The 5% threshold was chosen to identify the ~10-12 brain areas that contribute the most to the classification, resulting in better interpretability of the results.

**Clinical symptom prediction in females and males with ASD**

We investigated the relationship between stDNN identified neurobiological features (that distinguished between females and males with ASD) with the severity of clinical symptoms in females with ASD and males with ASD separately. The severity of clinical symptoms was assessed using ADI-R domain scores. The neurobiological features for each brain region were derived by computing the temporal median of the feature attribute weights which are obtained from the integrated gradients approach. The brain features were derived from the stDNN model that most accurately distinguished between females and males with ASD. To find the relationship between the clinical symptoms with these brain features, we computed Spearman correlations between the ADI-R RRB scores and the brain features derived from the best stDNN model. We applied FDR correction to correct for multiple comparisons. The aforementioned brain behavior analysis was repeated with ADI-R social and ADI-R communication scores. These analyses were conducted only on ABIDE/Stanford cohort data as ADI-R scores were not made available for the ASD participants in the CMI-HBN cohort.

**Control analysis examining the relationship between head movement and brain features underlying ASD sex classification**

We computed the squared distance correlation (dcor^2^) (30) between the strength of features and the mean framewise displacement (FD). Specifically, dcor^2^ between the strength of top 5% of features and mean FD was computed in the ASD female group for the two cohorts (ABIDE/Stanford and CMI-HBN) separately. Briefly, dcor^2^ is a measure of the nonlinear relationship between multidimensional variables, making it a better measure than conventional metrics like Pearson correlation, which only capture univariate linear relationships. dcor^2^ has a range from 0 to 1, with dcor^2^ = 0 denoting statistical independence.

Supplementary Results

**Results of fingerprint analysis**

We used an integrated gradients procedure (25-29) to compute the feature attributes underlying the ASD female class label in the ABIDE/Stanford cohort. This analysis yields a measure of feature strength associated with ASD females vs ASD males classification in each brain region and at each time point. The integrated gradients procedure was applied to the stDNN model that is trained to distinguish between ASD females and ASD males using ASD data from the ABIDE/Stanford cohort. This procedure also identifies an individual fingerprint of predictive features in each participant (**Figure 3**). We first examined whether these “fingerprints” cluster differently in females with ASD from males with ASD. Specifically, we computed a distance metric across brain features between individuals and compared the distances between individuals in females with ASD and males with ASD groups. We found that intra-female ASD group distance metrics were significantly shorter compared to distances to the male ASD group (*p* < 0.0001).

We then used the aforementioned procedures to determine predictive feature attributes in each ASD female participant in the CMI-HBN cohort. We found that intra-female ASD group distance metrics were significantly shorter compared to distances to the male ASD group (*p* < 0.0001), as in the Stanford/ABIDE cohort.

These results provide further proof of the highly accurate brain-based sex discrimination in ASD using stDNN.

**Results of control analyses to demonstrate the robustness of the main findings to head motion-related confounds**

First, we examined head motion using mean framewise displacement in the two groups. No significant differences were found in the mean framewise displacement between females with ASD and males with ASD (*p* = 0.44).

Second, we examined head motion using mean root mean square (RMS) in the two groups. No significant differences were found in the mean RMS between females with ASD and males with ASD (*p* = 0.58).

Third, we examined head motion using mean DVARS in the two groups. No significant differences were found in the mean DVARS between females with ASD and males with ASD (*p* = 0.37).

Fourth, we examined head motion using the percentage of scrubbed frames in the two groups. No significant differences were found in the percentage of scrubbed frames between females with ASD and males with ASD (*p* = 0.72).

Fifth, we examined head motion using mean framewise displacement in correctly and incorrectly classified ASD participants. We found that the correctly classified participants and incorrectly classified participants did not differ significantly in head motion parameters (*p* = 0.43).

Sixth, we added a scrubbing stage to our original preprocessing pipeline. The volumes to be excised were first identified using the recommended stringent criteria (FD > 0.2mm). The excised volumes were reestimated using interpolation. We applied our five-fold cross-validation procedure to this ‘scrubbed/interpolated’ dataset. Results of these additional analyses were very similar to the results from our original analysis: stDNN could distinguish between females with ASD and males with ASD with a high accuracy of 85.4 ± 2.07% and a precision of 0.84 ± 0.03, recall of 0.85 ± 0.02 and F1 score of 0.82 ± 0.03.

Seventh, we used the aCompCor-based nuisance signal estimation method in our preprocessing pipeline instead of the mean signal method. Specifically, we regressed out multiple nuisance signals from WM and CSF using PCA (no of PCs = 5). We applied our five-fold cross-validation procedure to this ‘aCompCor’ dataset. Results of these additional analyses were very similar to the results from our original analysis: stDNN could distinguish between females with ASD and males with ASD with a high accuracy of 85.0 ± 1.92% and a precision of 0.85 ± 0.02, recall of 0.85 ± 0.02 and F1 score of 0.82 ± 0.04.

Eighth, we excluded high motion participants. Specifically, we used a stringent exclusion criteria: >20% suprathreshold FDs, i.e., participants with more than 20% of their fMRI volumes with framewise displacement greater than 0.2 mm were excluded. Only 19 out of the 678 ASD participants were identified as high motion participants using this recommended criteria and were excluded. We applied our five-fold cross-validation procedure to this dataset (N_ASD = 659). Results of these additional analyses were very similar to the results from our original analysis: stDNN could distinguish between females with ASD and males with ASD with a high accuracy of 85.62 ± 1.3% and a precision of 0.85 ± 0.02, recall of 0.86 ± 0.01 and F1 score of 0.83 ± 0.01.

Ninth, we computed the squared distance correlation (dcor^2^) (30) between the strength of features and the mean framewise displacement (FD), and found no significant effect of head motion on the features (dcor^2^ = 0.08 ± 0.009). Briefly, dcor^2^ is a measure of the nonlinear relationship between multidimensional variables, making it a better measure than conventional metrics like Pearson correlation, which only capture univariate linear relationships. dcor^2^ has a range from 0 to 1, with dcor^2^ = 0 denoting statistical independence.

These results further attest to the robustness of our findings.

**Results of integrated gradients analysis to identify brain features underlying neurotypical** **sex classification**

We examined the feature attributes underlying the neurotypical female class label using an integrated gradients procedure. The integrated gradients procedure was applied to the stDNN model that is trained to distinguish between neurotypical females and neurotypical males using neurotypical data from the ABIDE/Stanford cohort. This analysis identified the posterior cingulate cortex, dorsolateral prefrontal cortex, frontopolar cortex, inferior temporal gyrus, thalamus, and ventral insula as the brain areas that contributed most significantly to predicting the neurotypical female class label. These brain areas are distinct from the brain areas that contributed most significantly to predicting the ASD female class label. These results are consistent with our classification analysis results that revealed that the stDNN model trained to distinguish between females with ASD and males with ASD could not distinguish between neurotypical females and neurotypical males, further demonstrating the unique pattern of brain sex differences in ASD that is different from normative/typical sex differences.

Supplementary Discussion

**Advantages of our XAI-based approach over previous approaches to find sex differences in ASD using functional brain imaging data**

To our knowledge, this is the first use of a XAI-based approach for uncovering robust neurobiological sex differences in ASD. Our quantitative XAI-based approach is a significant advance over previous approaches to find sex differences in ASD using functional brain imaging data. First, unlike previous static functional connectivity-based approaches, our XAI-based stDNN does not incorrectly assume the stationarity of fMRI timeseries and instead models the underlying non-stationarities in the data. Second, unlike previous univariate approaches, stDNN takes into account multivariate spatiotemporal patterns in the data, allowing it to detect subtle changes in multiple brain areas that may accompany complex neuropsychiatric disorders such as ASD, which would not be detectable by conventional univariate analysis. Third, unlike previous approaches agnostic to site-related data heterogeneity, stDNN incorporates multisite heterogeneity through one-hot-encoding to create a single model that handles heterogeneous data from different imaging protocols, yielding findings that are generalizable across sites, something that the previous approaches failed to achieve. Fourth, unlike extant DNN fully connected architectures, stDNN uses CNN and temporal averaging layers, making it particularly suited for training from a small number of participants, as is typically the case with fMRI data from neurodevelopmental populations. Fifth, unlike other DNN approaches, especially those applied to time series data, which are black box models that lack interpretability in terms of the underlying neurobiological features, the stDNN-based integrated gradients approach allows the computation of feature importance and consequently the identification of neurobiologically interpretable functional organization patterns that distinguish between groups. Lastly, unlike conventional machine learning methods, stDNN, through its use of LDAM loss function, is well suited for training datasets with class imbalance, as is typically the case with fMRI data from male-biased psychiatric conditions such as autism or female-biased conditions such as depression.

**Future work**

Further studies are needed to determine whether the sex differences identified in this study, which focused primarily on a sample of individuals on the autism spectrum who were high functioning as in extant studies, also exist in more severely affected individuals. Future work should also investigate how the observed sex differences in functional brain features relate to ADI-R RRB subscale scores including repetitive motor behaviors, insistence on sameness, and circumscribed interests, and/or other measures of RRB such as the Repetitive Behaviors Scale-Revised (RBS-R). Given our results that de novo trained models can distinguish between ASD females and males with greater accuracy than between neurotypical females and males, future research is needed to establish that sex has a stronger influence on neurobiology in ASD than neurotypicals. Future studies should also include relevant measures to delineate the direct and interactive effects of sex and gender on functional brain organization in ASD. In light of our findings that the brain systems that most significantly distinguish between ASD females and males primarily serve lateralized functions and previous evidence suggesting that biological sex impacts lateralization in brain systems (31), future studies are needed to investigate sex by group differences in brain asymmetry, and determine their differential links with clinical symptomology in females and males with ASD. Lastly, a larger harmonized longitudinal dataset covering a broad age range would be invaluable to make further assertions about the neurobiological sex differences in ASD observed here as well as its developmental trajectory.

Supplementary Tables

**Supplementary Table S1.** Demographic information for males and females with ASD and neurotypical males and females in the ABIDE/Stanford cohort.

|  | ASD Males (N=552) | ASD Females (N=126) | Neurotypical Males (N=639) | Neurotypical Females (N=337) | |
| --- | --- | --- | --- | --- | --- |
| **Age** | 13.1 ± 5.6 | 13.6 ± 6.9 | 13.9 ± 6.7 | 12.9 ± 6.4 |  |
| **ADI-R Social** | 17.9 ± 6.5 | 17.7 ± 7.0 |  |  |  |
| **ADI-R Verbal** | 14.6 ± 5.1 | 13.8 ± 5.5 |  |  |  |
| **ADI-R Restricted and Repetitive Behavior** | 5.6 ± 2.6 | 5.3 ± 2.7 |  |  |  |

**Supplementary Table S2.** Demographic information for males and females with ASD and neurotypical males and females in the CMI-HBN cohort.

|  | ASD Males (N=85) | ASD Females (N=10) | Neurotypical Males (N=59) | Neurotypical Females (N=49) |
| --- | --- | --- | --- | --- |
| **Age** | 11.9 ± 3.9 | 11.2 ± 3.7 | 10.7 ± 2.9 | 10.3 ± 2.6 |

**Supplementary Table S3.** ASD females vs. ASD males classification and five-fold cross-validation accuracy in ABIDE/Stanford cohort.

| Fold Number | Accuracy | Precision | Recall | F1-score |
| --- | --- | --- | --- | --- |
| Fold 1 | 88.7 | 0.89 | 0.89 | 0.87 |
| Fold 2 | 84.3 | 0.85 | 0.84 | 0.81 |
| Fold 3 | 86.0 | 0.85 | 0.86 | 0.84 |
| Fold 4 | 84.2 | 0.82 | 0.84 | 0.81 |
| Fold 5 | 86.7 | 0.89 | 0.87 | 0.82 |
| Avg | **86.0 ± 1.65 %** | **0.86 ± 0.02** | **0.86 ± 0.02** | **0.83 ± 0.02** |

**Supplementary Table S4.** ASD females vs. ASD males classification and five-fold cross-validation accuracy in the CMI-HBN cohort.

| Fold Number | Accuracy | Precision | Recall | F1-score |
| --- | --- | --- | --- | --- |
| Fold 1 | 82.1 | 0.84 | 0.82 | 0.83 |
| Fold 2 | 86.3 | 0.83 | 0.86 | 0.84 |
| Fold 3 | 77.9 | 0.85 | 0.78 | 0.81 |
| Fold 4 | 82.1 | 0.84 | 0.82 | 0.83 |
| Fold 5 | 88.4 | 0.87 | 0.88 | 0.87 |
| Avg | **83.4 ± 3.67 %** | **0.85 ± 0.01** | **0.83 ± 0.04** | **0.84 ± 0.02** |

**Supplementary Table S5.** Neurotypical females vs. neurotypical males classification and five-fold cross-validation accuracy in the ABIDE/Stanford cohort using the stDNN model trained to distinguish between ASD females and ASD males.

| Fold Number | Accuracy | Precision | Recall | F1-score |
| --- | --- | --- | --- | --- |
| Fold 1 | 67.2 | 0.65 | 0.67 | 0.62 |
| Fold 2 | 66.1 | 0.63 | 0.66 | 0.58 |
| Fold 3 | 67.9 | 0.66 | 0.68 | 0.63 |
| Fold 4 | 66.2 | 0.63 | 0.66 | 0.62 |
| Fold 5 | 66.1 | 0.63 | 0.66 | 0.57 |
| Avg | **66.7 ± 0.75 %** | **0.64 ± 0.01** | **0.67 ± 0.01** | **0.60 ± 0.02** |

**Supplementary Table S6.** Neurotypical females vs. neurotypical males classification and five-fold cross-validation accuracy in the ABIDE/Stanford cohort using the stDNN model trained to distinguish between neurotypical females and neurotypical males.

| Fold Number | Accuracy | Precision | Recall | F1-score |
| --- | --- | --- | --- | --- |
| Fold 1 | 77.7 | 0.77 | 0.78 | 0.77 |
| Fold 2 | 77.7 | 0.78 | 0.78 | 0.76 |
| Fold 3 | 78.5 | 0.78 | 0.78 | 0.78 |
| Fold 4 | 77.7 | 0.79 | 0.78 | 0.78 |
| Fold 5 | 77.2 | 0.77 | 0.77 | 0.75 |
| Avg | **77.8 ± 0.38 %** | **0.78 ± 0.01** | **0.77 ± 0.01** | **0.78 ± 0.01** |

**Supplementary Table S7.** Brain regions underlying ASD sex classification with the highest classification feature attributes (top 5%) in the ABIDE/Stanford cohort.

| **Brain Region** | **Subdivision** | **Brainnetome Atlas ID & Region Label** | **Feature Attribution Weights (10^-5^)** |
| --- | --- | --- | --- |
| Prefrontal Cortex | R IFJ, inferior frontal junction | ( 18 ), MFG_R_7_2 | 1.79 |
| Prefrontal Cortex | L A45r, rostral area 45 | ( 35 ), IFG_L_6_4 | 1.59 |
| Primary Motor | R A4hf, area 4 (head and face region) | ( 54 ), PrG_R_6_1 | 2.27 |
| Primary Motor | R A4ll, area 4 (lower limb region) | ( 68 ), PCL_R_2_2 | 1.84 |
| Temporal Gyrus | L A22r, rostral area 22 | ( 79 ), STG_L_6_6 | 2.28 |
| Temporal Gyrus | L A21c, caudal area 21 | ( 81 ), MTG_L_4_1 | 1.95 |
| Temporal Gyrus | R A20cl, caudolateral of area 20 | ( 100 ), ITG_R_7_6 | 1.70 |
| Parietal Cortex | L A5l, lateral area 5 | ( 129 ), SPL_L_5_3 | 1.79 |
| Parietal Cortex | R A5l, lateral area 5 | ( 130 ), SPL_R_5_3 | 1.72 |
| Parietal Cortex | L A7ip, intraparietal area 7 (hIP3) | ( 133 ), SPL_L_5_5 | 1.57 |
| Parietal Cortex | R A39rd, rostrodorsal area 39 (Hip3) | ( 138 ), IPL_R_6_2 | 1.64 |
| Occipital Gyrus | L mOccG, middle occipital gyrus | ( 199 ), LOcC_L_4_1 | 1.67 |
| Occipital Gyrus | R mOccG, middle occipital gyrus | ( 200 ), LOcC_R_4_1 | 1.61 |

**Supplementary Table S8.** Brain regions underlying ASD sex classification with the highest classification feature attributes (top 5%) in the CMI-HBN cohort.

| **Brain Region** | **Subdivision** | **Brainnetome Atlas ID & Region Label** | **Feature Attribution Weights (10^-5^)** |
| --- | --- | --- | --- |
| Prefrontal Cortex | R A45c, caudal area 45 | ( 34 ), IFG_R_6_3 | 2.99 |
| Premotor | R A6cvl, caudal ventrolateral area 6 | ( 64 ), PrG_R_6_6 | 2.76 |
| Primary Motor | L A4ll, area 4 (lower limb region) | ( 67), PCL_L_2_2 | 3.59 |
| Primary Motor | R A4ll, area 4 (lower limb region) | ( 68 ), PCL_R_2_2 | 5.54 |
| Temporal Gyrus | R A22c, caudal area 22 | ( 76 ), STG_R_6_4 | 2.86 |
| Temporal Gyrus | L A21c, caudal area 21 | ( 81 ), MTG_L_4_1 | 4.63 |
| Temporal Gyrus | R A20cl, caudolateral of area 20 | ( 100 ), ITG_R_7_6 | 3.20 |
| Parahippocampal Gyrus | R TH, area TH (medial PPHC) | ( 120 ), PhG_R_6_6 | 3.05 |
| Parietal Cortex | R A5l, lateral area 5 | ( 130 ), SPL_R_5_3 | 2.87 |
| Parietal Cortex | R A39rd, rostrodorsal area 39 (Hip3) | ( 138 ), IPL_R_6_2 | 4.38 |
| Mid Cingulate Cortex | L A24rv, rostroventral area 24 | ( 177 ), CG_L_7_2 | 4.67 |
| Posterior Cingulate Cortex | R A23c, caudal area 23 | ( 186 ), CG_R_7_6 | 2.98 |
| Occipital Gyrus | L mOccG, middle occipital gyrus | ( 199 ), LOcC_L_4_1 | 3.18 |

Supplementary Figures

**Supplementary Figure S1.** For each subject, the stDNN model uses regional fMRI timeseries and covariates including site as input. The model predicts the class label (female with ASD or male with ASD) of the subject using spatiotemporal convolutions of the fMRI timeseries along with the covariate information. The integrated gradients of the model’s prediction to its input is used for identifying “black-box” brain features underlying the classification of females with ASD versus males with ASD.

**
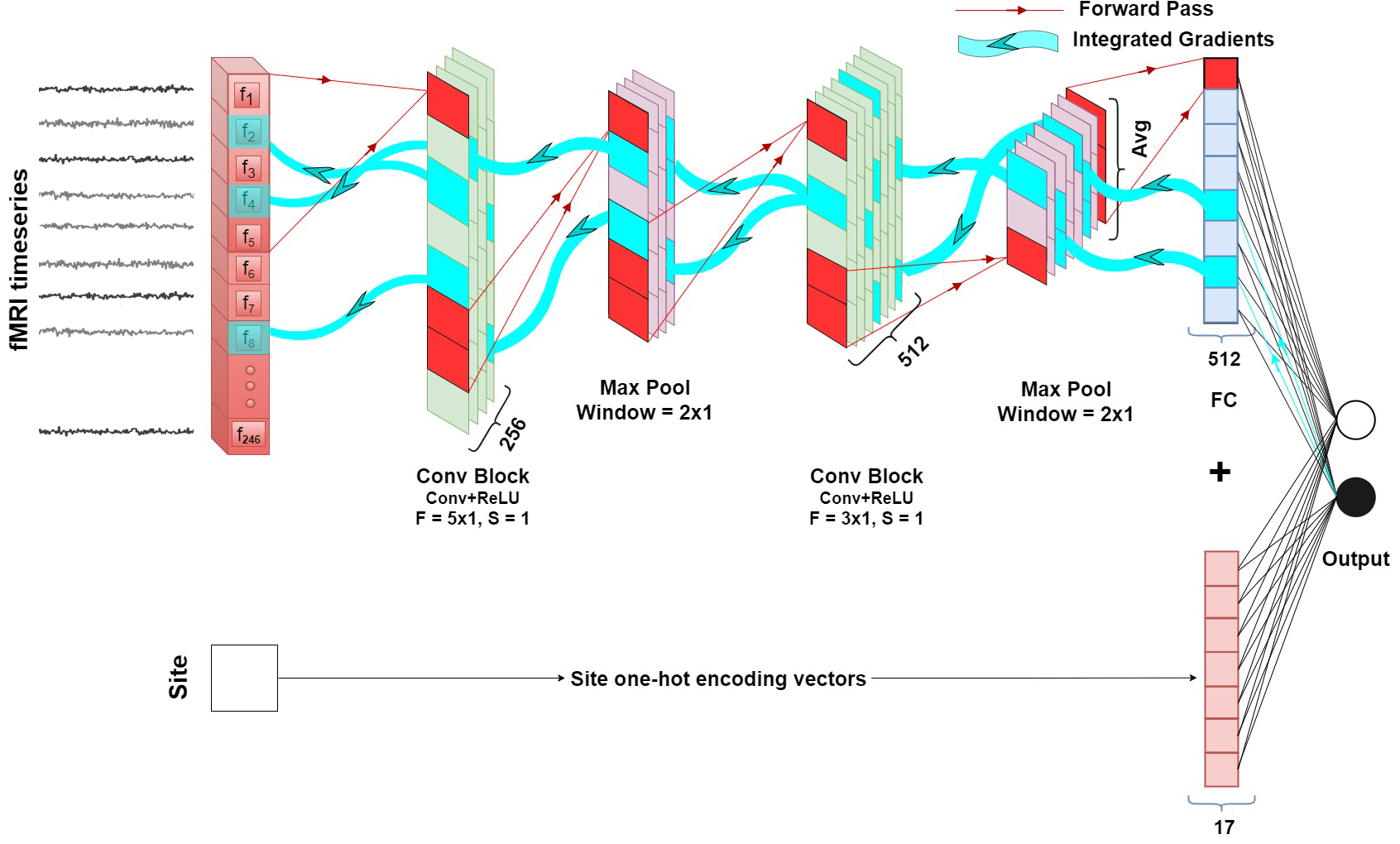
**

**Supplementary Figure S2. Participant selection procedure for the ABIDE cohort.**

**
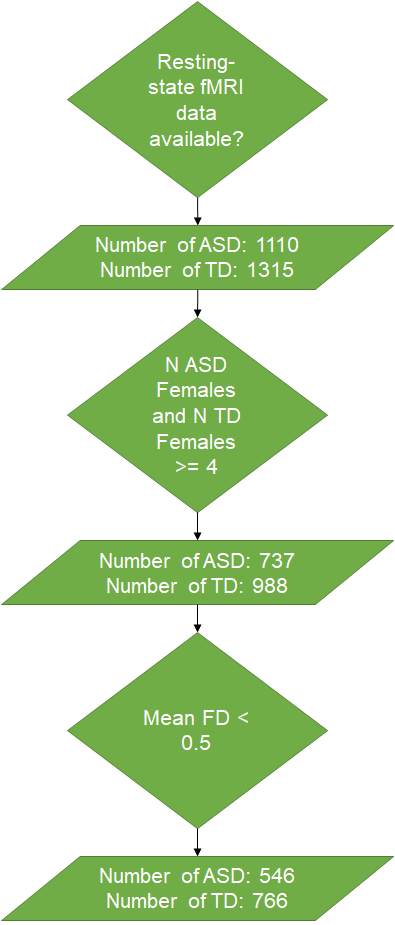
**

**Supplementary Figure S3. Participant selection procedure for the Stanford cohort.**

**
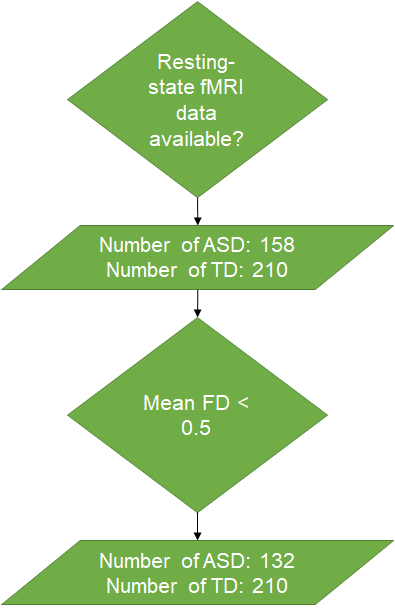
**

**Supplementary Figure S4. Participant selection procedure for the CMI-HBN cohort.**

**
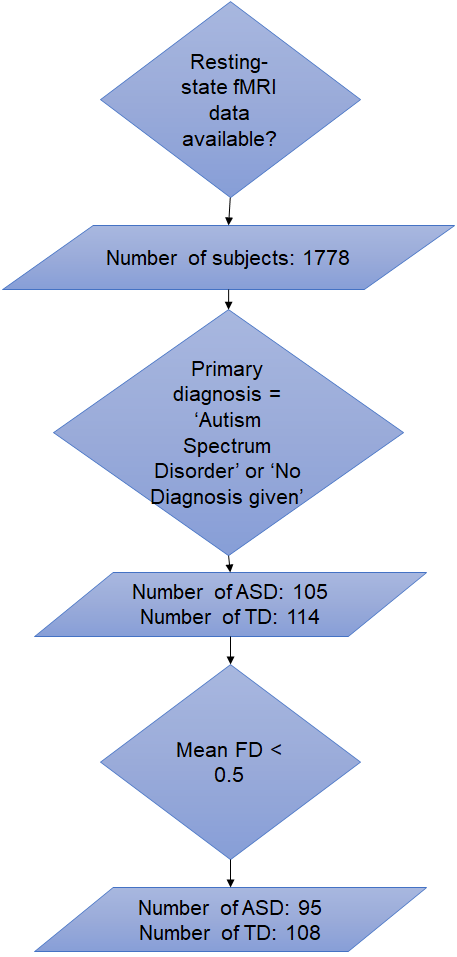
**

Supplementary References

1. D. Durstewitz, G. Koppe, A. Meyer-Lindenberg, Deep neural networks in psychiatry. *Mol Psychiatry* **24**, 1583-1598 (2019).

2. M. J. M. Walsh, G. L. Wallace, S. M. Gallegos, B. B. Braden, Brain-based sex differences in autism spectrum disorder across the lifespan: A systematic review of structural MRI, fMRI, and DTI findings. *Neuroimage Clin* **31**, 102719 (2021).

3. A. N. V. Ruigrok, M. C. Lai, Sex/gender differences in neurology and psychiatry: Autism. *Handb Clin Neurol* **175**, 283-297 (2020).

4. J. Kim, V. D. Calhoun, E. Shim, J. H. Lee, Deep neural network with weight sparsity control and pre-training extracts hierarchical features and enhances classification performance: Evidence from whole-brain resting-state functional connectivity patterns of schizophrenia. *Neuroimage* **124**, 127-146 (2016).

5. S. Ryali *et al.*, Temporal Dynamics and Developmental Maturation of Salience, Default and Central-Executive Network Interactions Revealed by Variational Bayes Hidden Markov Modeling. *PLoS Comput Biol* **12**, e1005138 (2016).

6. J. Taghia *et al.*, Uncovering hidden brain state dynamics that regulate performance and decision-making during cognition. *Nat Commun* **9**, 2505 (2018).

7. S. Ryali, K. Supekar, T. Chen, V. Menon, Multivariate dynamical systems models for estimating causal interactions in fMRI. *Neuroimage* **54**, 807-823 (2011).

8. G. Koppe, A. Meyer-Lindenberg, D. Durstewitz, Deep learning for small and big data in psychiatry. *Neuropsychopharmacology* **46**, 176-190 (2021).

9. V. D. Calhoun, R. Miller, G. Pearlson, T. Adali, The chronnectome: time-varying connectivity networks as the next frontier in fMRI data discovery. *Neuron* **84**, 262-274 (2014).

10. W. Cai, T. Chen, L. Szegletes, K. Supekar, V. Menon, Aberrant Time-Varying Cross-Network Interactions in Children With Attention-Deficit/Hyperactivity Disorder and the Relation to Attention Deficits. *Biol Psychiatry Cogn Neurosci Neuroimaging* **3**, 263-273 (2018).

11. K. Supekar, W. Cai, R. Krishnadas, L. Palaniyappan, V. Menon, Dysregulated Brain Dynamics in a Triple-Network Saliency Model of Schizophrenia and Its Relation to Psychosis. *Biol Psychiatry* **85**, 60-69 (2019).

12. A. Di Martino *et al.*, The autism brain imaging data exchange: towards a large-scale evaluation of the intrinsic brain architecture in autism. *Mol Psychiatry* **19**, 659-667 (2014).

13. A. Di Martino *et al.*, Enhancing studies of the connectome in autism using the autism brain imaging data exchange II. *Sci Data* **4**, 170010 (2017).

14. K. Supekar, S. Ryali, P. Mistry, V. Menon, Aberrant dynamics of cognitive control and motor circuits predict distinct restricted and repetitive behaviors in children with autism. *Nat Commun* **12**, 3537 (2021).

15. K. Supekar *et al.*, Brain hyperconnectivity in children with autism and its links to social deficits. *Cell Rep* **5**, 738-747 (2013).

16. D. A. Abrams *et al.*, Impaired voice processing in reward and salience circuits predicts social communication in children with autism. *Elife* **8** (2019).

17. D. A. Abrams *et al.*, Underconnectivity between voice-selective cortex and reward circuitry in children with autism. *Proc Natl Acad Sci U S A* **110**, 12060-12065 (2013).

18. C. J. Lynch *et al.*, Default mode network in childhood autism: posteromedial cortex heterogeneity and relationship with social deficits. *Biol Psychiatry* **74**, 212-219 (2013).

19. D. O'Connor *et al.*, The Healthy Brain Network Serial Scanning Initiative: a resource for evaluating inter-individual differences and their reliabilities across scan conditions and sessions. *Gigascience* **6**, 1-14 (2017).

20. L. Fan *et al.*, The Human Brainnetome Atlas: A New Brain Atlas Based on Connectional Architecture. *Cereb Cortex* **26**, 3508-3526 (2016).

21. C. Davatzikos, Machine learning in neuroimaging: Progress and challenges. *Neuroimage* **197**, 652-656 (2019).

22. D. P. Kingma, J. Ba, Adam: A method for stochastic optimization. *Proceedings of the 3rd International Conference on Learning Representations (ICLR)* **1412** (2015).

23. K. Cao, C. Wei, A. Gaidon, N. Arechiga, T. Ma, Learning imbalanced datasets with label-distribution-aware margin loss. *arXiv preprint arXiv:1906.07413* (2019).

24. R. Liaw *et al.*, Tune: A research platform for distributed model selection and training. *arXiv preprint arXiv:1807.05118* (2018).

25. S. M. Lundberg, S.-I. Lee (2017) A unified approach to interpreting model predictions. in *Advances in neural information processing systems*, pp 4765-4774.

26. K. Simonyan, A. Vedaldi, A. Zisserman, Deep inside convolutional networks: Visualising image classification models and saliency maps. *arXiv preprint arXiv:1312.6034* (2013).

27. J. T. Springenberg, A. Dosovitskiy, T. Brox, M. Riedmiller, Striving for simplicity: The all convolutional net. *arXiv preprint arXiv:1412.6806* (2014).

28. R. R. Selvaraju *et al.* (2017) Grad-cam: Visual explanations from deep networks via gradient-based localization. in *Proceedings of the IEEE international conference on computer vision*, pp 618-626.

29. M. Sundararajan, A. Taly, Q. Yan (2017) Axiomatic attribution for deep networks. in *Proceedings of the 34th International Conference on Machine Learning-Volume 70* (JMLR. org), pp 3319-3328.

30. G. J. Székely, M. L. Rizzo, N. K. Bakirov, Measuring and testing dependence by correlation of distances. *The annals of statistics* **35**, 2769-2794 (2007).

31. H. Liu, S. M. Stufflebeam, J. Sepulcre, T. Hedden, R. L. Buckner, Evidence from intrinsic activity that asymmetry of the human brain is controlled by multiple factors. *Proc Natl Acad Sci U S A* **106**, 20499-20503 (2009).
